# Dynamics and energetics of PCBP1 binding to severely oxidized RNA

**DOI:** 10.3389/fmolb.2022.994915

**Published:** 2022-11-03

**Authors:** Natacha Gillet, Elise Dumont

**Affiliations:** ^1^ Laboratoire de Chimie, ENS de Lyon, CNRS UMR 5182, Lyon, France; ^2^ CNRS, Institut de Chimie de Nice, Université Côte d’Azur, Nice, France; ^3^ Institut Universitaire de France, Paris, France

**Keywords:** RNA-binding protein, 8-oxoguanine, damage recognition, molecular dynamics simulations, conformational analysis

## Abstract

Oxidatively generated lesions such as 8-oxo-7, 8-dihydroguanine (8-oxoG) on RNA strands constitute a hallmark marker of the oxidative stress in the cell. Poly-C binding protein 1 (PCBP1) is able to specifically recognize severely damaged RNA strands containing two 8-oxoG lesions separated by five nucleobases, which trigger a signaling pathway leading to cell apoptosis. We apply an *in silico* protocol based on microsecond timescale all-atom classical molecular dynamics simulations associated with conformational and energy analyses to unveil the specific recognition mechanism at a molecular level. By comparing the RNA and protein behavior for sequences with six different damage profiles, our results highlight an allosteric mechanism, allowing a stronger binding of the oxidized guanine at position 9 only if another 8-oxoG lesion is present at position 15, in full agreement with experiments. We assess the role of lysine K23 and the additional ketone group of the oxidized guanine, thanks to computational site-directed mutagenesis.

## Introduction

Guanine (G) is one of the four constitutive nucleobases of RNA, which pairs up with cytosine. It singles out by the existence of stable G-quadruplexes (G4) and its relatively easy oxidation as it features the lowest ionization amongst the canonical RNA/DNA nucleobases. Oxidation of guanine mainly results in 8-oxo-7,8-dihydroguanine (8-oxoguanine or 8-oxoG or °G), which is prone to pair not only with cytosine but also with adenine, conferring to this ubiquitous oxidatively induced nucleobase a potentially deleterious nature. Moreover, the presence of an additional ketone on the purine ring can modify the conformation of the DNA backbone (Dršata et al., 2013; Hoppins et al., 2016; Kara and Zacharias, 2013). Then, 8-oxoG can evolve toward secondary products which also impede the correct DNA transcription of replications (Cadet et al., 2017). As other DNA damages, 8-oxoG is recognized by different proteins to call for DNA repair machinery (Poetsch, 2020; Popov et al., 2020).

Single strand RNA is much more flexible than DNA, and the recognition of the damages does not involve the same proteins and the same mechanism as the well-shaped double DNA strand. It is also less crucial to repair RNA than DNA with regard to the replication of the genetic information, although mRNA oxidation can impact translation processes (Tanaka and Chock, 2021; Yan and Zaher, 2019) and be associated with various diseases, such as neurodegenerative diseases (Li et al., 2020). To avoid such consequences, the oxidized RNA can be degraded, while the recognition of severely damaged RNA can be an indicator of high exposure to oxidative stress and can, thus, trigger signaling pathways that can result in cell apoptosis (Ishii and Sekiguchi, 2019).

**FIGURE 1:**
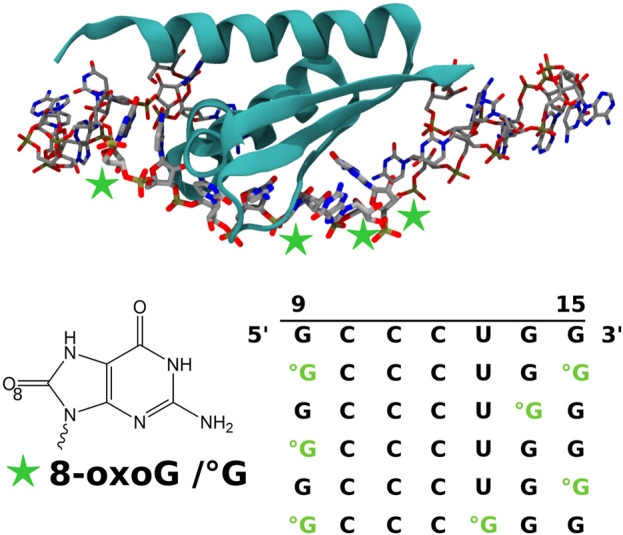
Representation of the KH 1 domain of the PCBP1 protein in the interaction with the 24-nucleobase RNA strands obtained after RNA sequence modification of the 3VKE PDB structure (Yoga et al., 2012). The considered positions of the 8-oxoguanine (8-oxoG/°G) lesion are indicated by green stars. The obtained control and modified sequences for the nucleobases of positions 9 to 15 are also given. All biomolecule pictures have been rendered using VMD (Humphrey et al., 1996).

Indeed, it has been experimentally suggested that PCBP1 binding to oxidized RNA (Figure 1) consists in the first step of one of such pathways (Ishii et al., 2018). Unlike the recognition of the damaged RNA by AUF1, which leads to the degradation of the oxidized strand, RNA binding by PCBP1 activates the same apoptosis reaction as the recognition of DNA oxidative damages, caspase-3 activation, and PARP-1 cleavage. To the best of our knowledge, the interaction mechanisms of the factors bound to the RNA-PCBP1 complex remain unknown. PCPB1 belongs to a protein family that binds poly-cytosine strands and includes PCBP1, PCBP2, PCBP3, and PCBP4. All of them can interact with RNA strands, thanks to the highly conserved KH 1 domain, but only PCBP1 and PCBP2 show a preference for sequences rich in 8-oxoG (Ishii et al., 2020). However, the consequences of this recognition differ as PCBP1 increases apoptosis, whereas PCBP2 seems to impede it. This competition between apoptosis and survival of highly oxidized cells can be at play in tumor cell occurrence. Interestingly, this recognition is quite specific and requires the presence of two 8-oxoG lesions spaced by five nucleobases (see sequences in Figure 1). Even though experiments have proven that one double mutant (K31D and K32D) hinders RNA binding (Ishii et al., 2018), the molecular mechanism of the damaged RNA recognition remains unknown.

In this work, we scrutinize the PCBP1 KH 1 domain behavior in the presence of the RNA sequence with different oxidized spots by means of *in silico* all-atom molecular dynamics (MD) simulations. Recently, our simulation and analysis protocol has successfully revealed the changes in the interaction network between a glycosylase and a DNA strand containing an 8-oxoG or a tandem lesion (Bignon et al., 2021; Jiang et al., 2021). Indeed, the combination of microsecond timescale classical MD simulations and various analysis approaches, including machine learning post-processing, allows the detection of slight conformational modifications which, once combined, can induce a dramatic change in the recognition and binding mechanism. Here, we also take advantage of the *in silico* tools to test not only different RNA sequences but also protein mutants to computationally assess the role of some residues, which is of a lower cost than the corresponding experiment.

## Methods

We started from the PCBP1 KH 1 domain structure in interaction with poly-C RNA (5′-ACCCCA-3′) (PDB code 3VKE; Yoga et al., 2012). We modified the RNA sequence within the interacting part (replacing CCCC by CCCU) and by adding nucleobases to obtain the 5′-UGG​CCA​AU**GCC​CUG​G**CUC​ACA​AAU-3′ RNA sequence using the chimera RNA builder (Pettersen et al., 2004). The nucleobases were modified to the 8-oxoG lesion in agreement with the following aimed sequences between positions 9 and 15: °GCCCUG°G, GCCCU°GG, °GCCCUGG, GCCCUG°G, and °GCCC°GGG. All the protein–RNA complexes were solvated in a cubic TIP3P (Jorgensen et al., 1983) box of 95-Å side length. NaCl salt was added to neutralize the box and reach an ionic strength of 0.15 M. The Amberff14sb (Maier et al., 2015), parmbsc0+ol3 (Pérez et al., 2007; Zgarbová et al., 2011), and GAFF (Wang et al., 2006) force field parameters have been used for protein, RNA, and 8-oxoG residues, respectively. All simulations were performed using the Amber18 (Case, 2018) suite of programs. After minimization, each system was heated from 0 to 300 K during 30 ps (time step of 1 fs), and a short equilibration (1 ns, time step of 2 fs with the SHAKE algorithm, 300 K, 1 bar) was performed before a 1-*μ*s production run with identical parameters. Periodic boundary conditions and particle mesh Ewald were used for all simulations, and the non-bonded cutoff was fixed at 12 Å. Trajectory convergence was checked by following the root-mean-square deviations (RMSD) of the protein and 9 to 15 RNA sequences (see Supplementary Figures S1A, B). Protein mutations K23Q, K23 M, and K31D were proceeded on the final geometry of the WT simulation in the presence of the °GCCCUG°G sequence. Then, the modified systems were equilibrated and simulated, following the previously described protocol (see Supplementary Figures S1C, D). Distances, RMSD, and radial distribution functions were obtained using the CPPTRAJ module (Roe and Cheatham, 2013), MMPBSA binding free energies using the MMPBSA module from Amber18 (Miller et al., 2012), and RNA structural parameters using the Curves + program (Lavery et al., 2009). Unsupervised machine learning-based principal component analysis was performed using our home program (Bignon et al., 2020). In addition, the dynamic cross correlation between the residues for the control and the °GCCCUG°G systems was obtained using the Bio3D R library (Grant et al., 2021). All heavy atoms of the protein and the 9 to 15 RNA residues were taken into account to determine the dynamic cross correlations along the 1-*μ*s trajectories which are then averaged per residue. From this, a community network is created by clustering the residues with a similar cross correlation pattern, revealing nodes of connected residues. A cutoff of 0.8 is used on the cross-correlation matrix values. The protein structure network community can be visualized with a VMD molecular viewer (Humphrey et al., 1996).

## Results

### RNA damaged sequence binding

An RNA single strand explores a large conformational space due to its high flexibility (Hagerman, 1997). In our trajectories, we observe that the CCC sequence is kept strongly bound to the PCBP1 protein, regardless of the presence of the damage, ensuring proximity of the whole sequence with the PCBP1 KH 1 domain, especially for the residues 9 to 15. Then, the 1 to 8 and 16 to 24 parts of the RNA strand remain free to move and quickly fold around the protein surface. This conformational landscape is not relevant as the shape of the biological protein certainly differs from the one of our simulations, where KH 2 and KH 3 domains are missing. Consequently, we will focus our analysis on the behavior of the nucleobases between positions 9 and 15 and their interaction with the present amino acids.

First, we question the impact of the presence of an 8-oxoG lesion damage on the RNA structures. The six inter-nucleobase parameters (shift, slide, rise, tilt, roll, and twist) and backbone dihedral angles are reported in Supplementary Figures S2,S3. The 10–11 and 11–12 pairs (corresponding to the common CCC sequence) have a similar behavior, regardless of the sequence, with relatively small fluctuations, consistent with a strong binding to the protein. Except when the uracil in position 13 is replaced by an 8-oxoG lesion, the 12–13 pair also follows the same structural behavior, suggesting a conformational stabilization by protein residues. The 9–10 pair conformation fluctuates more, especially for the GCCCUG°G sequence where the guanine in position 9 falls in a different geometry compared to the other systems. The 13–14 base pair behavior differs more when a damaged guanine is present in position 13 or 14, whereas the most diverse inter-base profile corresponds to the 14–15 base pair, thanks to a larger conformational space explored by the guanine at position 15, which is less constrained by the protein. Overall, the standard deviations for the °GCCCUG°G inter-base parameters are smaller than those for the other sequences, suggesting a more rigid strand. The backbone dihedral angles corroborate these conclusions, with common behaviors observed for CCC sequences (10, 11, and 12 nucleobases). Interestingly, the *χ* angle for 8-oxoG seems to be constrained as the associated standard deviations are relatively smaller than those associated with a guanine at the same position.

**FIGURE 2:**
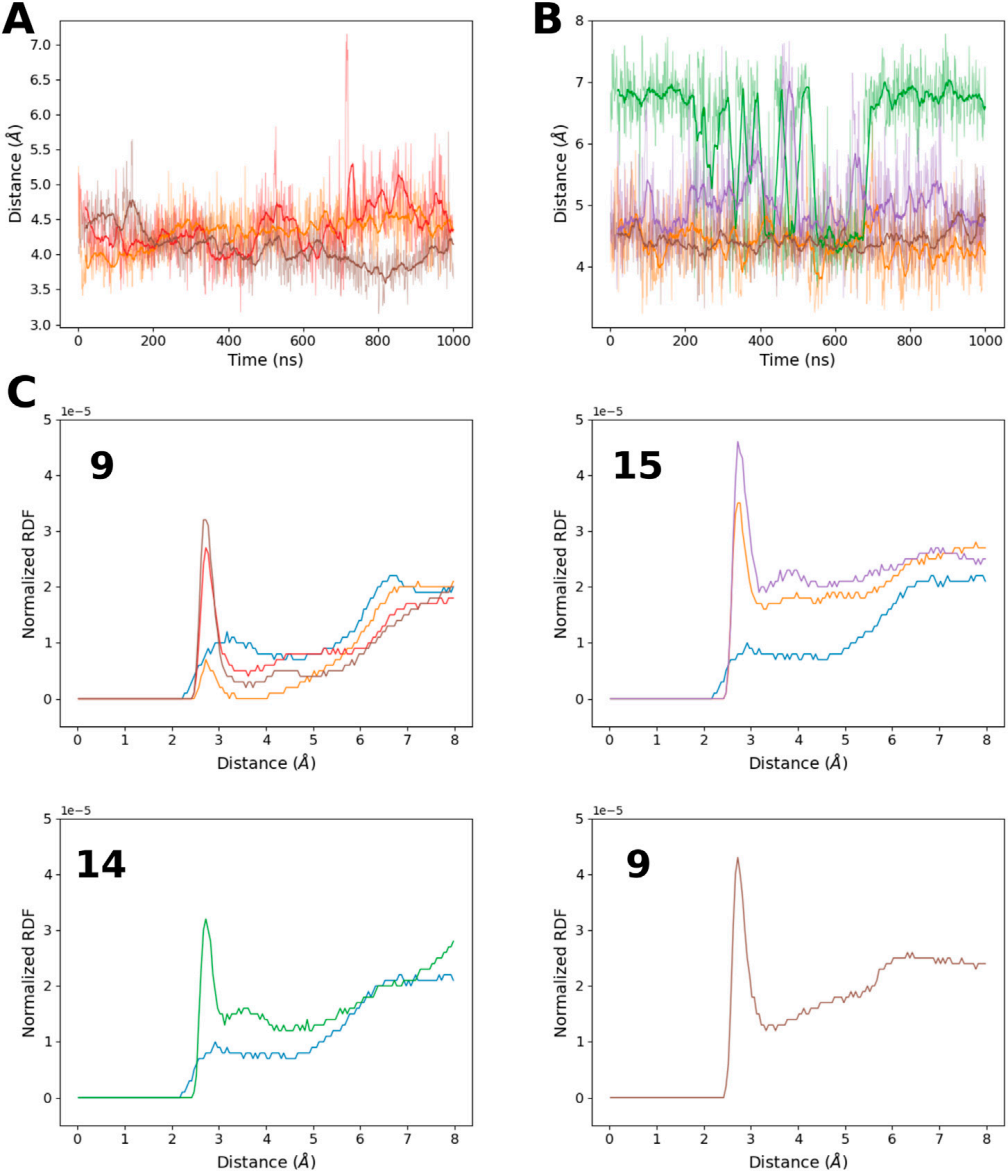
**(A)** Time evolution of the distance between 8-oxoG9 O8 and the closest 8-oxoG9 phosphate oxygen atom for the RNA sequences: °GCCCUG°G (orange), °GCCCUGG (red), and °GCCC°GGG (brown). **(B)** Time evolution of the distance between 8-oxoG O8 and the closest 8-oxoG phosphate oxygen atom for damage at position 15 in the RNA sequences °GCCCUG°G (orange) and GCCCUG°G (purple), at position 14 for the RNA sequence GCCCU°GG (green), or at position 13 for °GCCC°GGG (brown). **(C)** Radial distribution function of the water oxygen atom around the O8 atom of different 8-oxoG lesions in position 9, 15, 14, or 13. If possible, the corresponding radial distribution function around the H8 atom of the undamaged guanine in the control simulation is also represented. Line colors correspond to the RNA sequences: control (blue), °GCCCUG°G (orange), GCCCU°GG (green), °GCCCUGG (red), GCCCUG°G (violet), and °GCCC°GGG (brown).

Indeed, the distance between the 8-oxoG O8 atoms and the phosphate oxygen atoms seems to be restrained (Figures 2A,B **)** around 4 Å , regardless of the damage position or the previously described RNA structural parameters. As the two oxygen atoms cannot have a direct attractive interaction due to their chemical nature, this conformational constraint must involve another polar molecule. The radial distribution function of the water oxygen atoms around O8 (Figure 2C) indicates the presence of a mediating water molecule with a peak centered at 2.7 Å  consistent with a hydrogen bond and absent in the control sequence simulation (selecting H8 instead of O8). Surprisingly, this water molecule also seems to be absent around the 8-oxoG9 lesion of the °GCCCUG°G sequence, whereas the O8–phosphate distance is maintained at around 4 Å. The proximity of protein residues can explain this behavior.

We have determined the binding energy of the RNA–PCBP1 complex using the MMPBSA approach for the different sequences at different times of the trajectories (see Table 1). We observe an attractive binding energy of about 25 kcal/mol when 8-oxoGs are present at positions 9 and 15, so an increase of at least 12 kcal/mol compared to the undamaged control sequence. This energy decreases when only one damage is present on the sequence from 6 kcal/mol (for 8-oxoG in position 14, which can also present a 7 kcal/mol stronger binding energy) to 22 kcal/mol (for 8-oxoG in position 15). Putting the damages closer, at positions 9 and 13, decreases the binding energy by about 18–25 kcal/mol. All these energy differences must be tempered due to the large standard deviation of 8–11 kcal/mol and the trend of the MMPBSA approach to overestimate the binding energy. Nevertheless, it clearly appears that the interaction between the PCBP1 KH 1 domain and RNA is better for the °GCCCUG°G sequence in our simulations, in agreement with the experiment (Ishii et al., 2018).

**TABLE 1:** Average and standard deviations (in kcal/mol) of the binding energy of the RNA fragment GCCCUGG to the PCBP1 RNA-binding domain for the different oxidized guanine positions. The values are determined at different times along the 1-*μ*s trajectories.

Time (*μ*s)	Control	°GCCCUG°G	GCCCU°GG	°GCCCUGG	GCCCUG°G	°GCCC°GGG
0.5–0.6	−2.1 ± 8.9	−26.4 ± 8.4	−33.2 ± 11.9	−16.1 ± 7.3	−11.7 ± 8.1	0.4 ± 12.2
0.7–0.8	−12.8 ± 8.9	−24.8 ± 7.3	−19.3 ± 9.3	−8.1 ± 7.8	−10.5 ± 8.4	−3.9 ± 10.6
0.9–1	−13.3 ± 7.7	−25.5 ± 8.1	−19.6 ± 10.9	−10.0 ± 8.7	−3.3 ± 10.6	−7.2 ± 10.6

**FIGURE 3:**
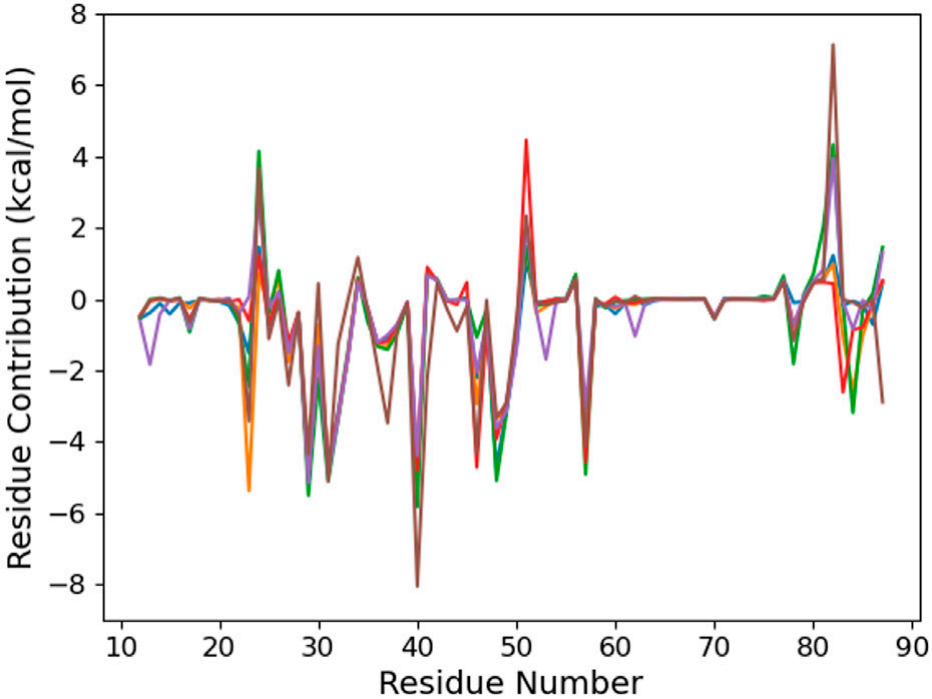
Per-protein residue contribution to the binding energy of the RNA fragment for the six studied RNA sequences: control (blue), °GCCCUG°G (orange), GCCCU°GG (green), °GCCCUGG (red), GCCCUG°G (violet), and °GCCC°GGG (brown).

**FIGURE 4:**
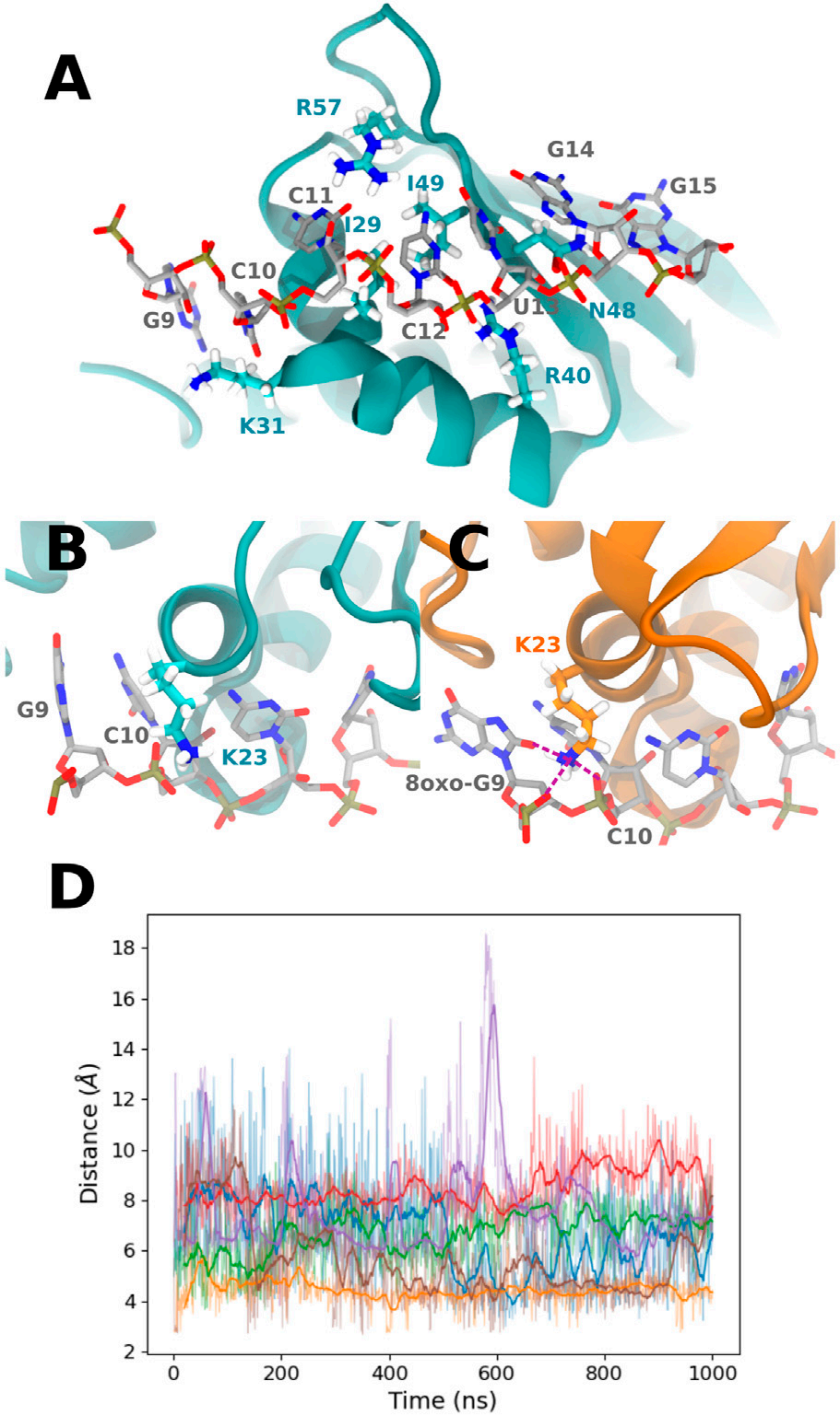
**(A)** Representation of the PCBP1 residues interacting with all RNA sequences, mostly with nucleobases at positions 10 to 14 (from the control sequence in this picture). **(B)** Example of K23 conformation in the presence of the control sequence. **(C)** K23 interaction with 8-oxoG9 O8 and 8-oxoG9 and C10 phosphate in the presence of the °GCCCUG°G sequence. Interactions are represented with purple dashed lines. **(D)** Time evolution of the distance between 8-oxoG9 O8 and the K23 N*ζ* atom for the six studied RNA sequences: control (blue), °GCCCUG°G (orange), GCCCU°GG (green), °GCCCUGG (red), GCCCUG°G (violet), and °GCCC°GGG (brown).

To provide a molecular explanation to this preference, we first consider the per-residue contribution to the binding energy (Figure 3). Six residues—I29, K31, R40, N48, I49, and R57—contribute in a similar way to RNA binding, regardless of the damage presence or position. Actually, they interact with the CCCUG common sequence (see Figure 4A). Some carboxylates, E51 and D82, weaken the RNA–protein interaction by a varying range with regards to the sequence. Actually, these residues present a dynamical behavior, and from time to time, they come close to the RNA bases and their oxygen atoms. These conformations are present in the last 100 ns of some trajectories, corresponding to the positive peaks, but can be absent earlier in the simulation or observed for other systems at a different time.

The contribution of K23 is about −5.3 kcal/mol for the °GCCCUG°G sequence, while this residue can interact with the 8-oxoG lesion in position 9. Intriguingly, this lysine also stabilizes the interaction with RNA to a lesser extent not only for the °GCCC°GGG sequence but also for the GCCCU°GG sequence, whereas the ninth nucleobase is not oxidized. For these two sequences, this stabilization is counterbalanced by the positive contribution of E24. On the contrary, K23 does not interact with the 8-oxoG lesion of the one damaged °GCCCUGG sequence. Indeed, in the 9–15th damaged sequence, K23 reaches a conformation where its ammonium group stands between the O8 atom of 8-oxoG and the phosphates of nucleobases at positions 9 and 10 (see Figures 4B–D). This interaction starts after about 300 ns and is present until the end of the simulation. A similar interaction is transiently observed in the presence of the °GCCC°GGG sequence. For the GCCCU°GG sequence, K23 does not reach the RNA backbone but can interact with O6 of the guanine at position 9.

Our results highlight a better stabilization of the 8-oxoG lesion than the undamaged guanine, especially for the °GCCCUG°G sequence. Surprisingly, if this observation is supported by stabilizing interactions around the 8-oxoG lesion at position 9, no protein residue is identified as contributing to the binding around position 15. Nevertheless, the comparison with the °GCCCUGG sequence strongly suggests that both damages are required to strengthen the RNA–protein complex. Consequently, we explore the hypothesis of a conformational communication along the RNA-binding domain to explain the cooperative role of the two 8-oxoG lesions.

**FIGURE 5:**
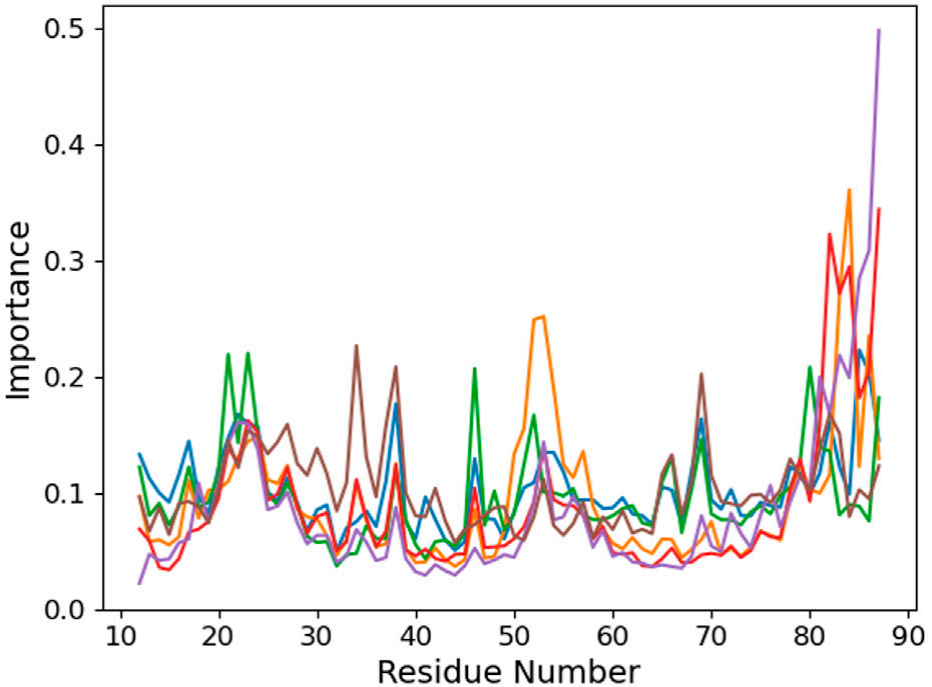
Per-protein residue importance from the PCA analysis for the six studied RNA sequences: control (blue), °GCCCUG°G (orange), GCCCU°GG (green), °GCCCUGG (red), GCCCUG°G (violet), and °GCCC°GGG (brown).

We use an unsupervised machine learning approach based on principal component analysis (PCA) to discriminate the importance of the different protein residues with respect to the binding conformation (Figure 5). Focusing on the °GCCCUG°G sequence, we observe an interesting peak in the importance for the 49–55 region of the protein which is absent in the other systems. This part of the protein corresponds to a loop linking two *β*-sheet strands. Three threonines of this *β*-sheet are close to the RNA 15th nucleobase (T15, T60, and T62), while the loop itself is relatively close to the positions 9 to 12 and to K23.

**FIGURE 6:**
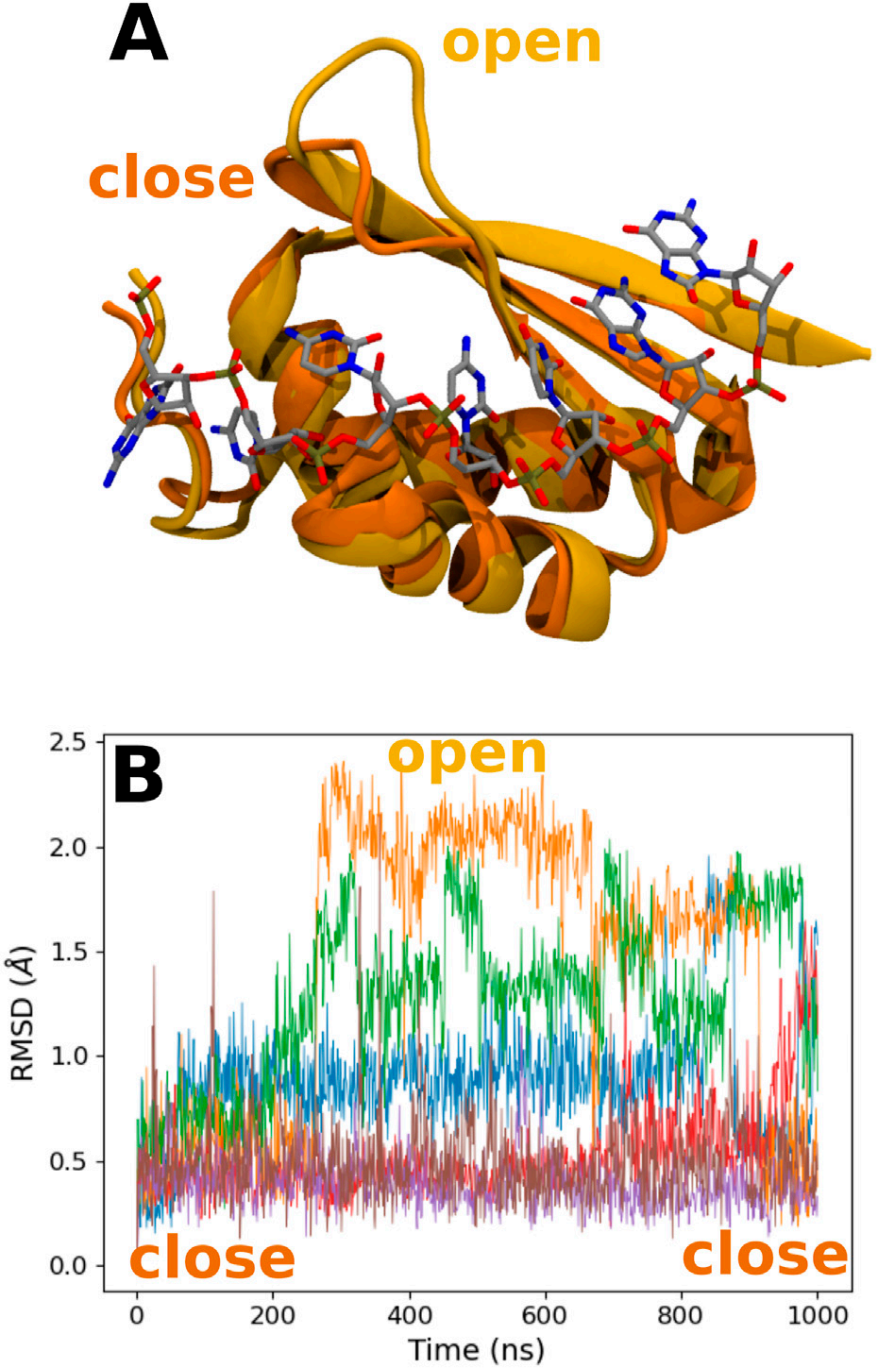
**(A)** Representation of the close and open conformations of the 49–55 PCBP1 loop in the presence of the °GCCCUG°G sequence. **(B)** RMSD of the C*α* atoms of the 49–55 PCBP1 residues in the presence of the six studied RNA sequences: control (blue), °GCCCUG°G (orange), GCCCU°GG (green), °GCCCUGG (red), GCCCUG°G (violet), and °GCCC°GGG (brown).

Looking at the trajectory, we observe a motion of the 49–55 loop occurring after 250 ns (see Figure 6). This motion allows the loop to reach a kind of open conformation, and at the same time, K23 is able to come close to the RNA backbone and interact with the O8 atom of 8-oxoG and the backbone, as described previously. This occurs simultaneously with the formation of transient hydrogen bonds between 8-oxoG15 N1 and N2 and the T15 alcohol group, thereby with a close conformation of the damaged guanine toward the *β*-sheet (see Supplementary Figure S4B). When the loop is open, hydrogen bonds are formed between S50 and G14 N1 and O6 (see Supplementary Figure S4E). At the end of the trajectory, the loop recovers a close position, but K23 keeps its binding conformation. This motion of the tail is only observed for the °GCCCUG°G sequence, even though a slightly open conformation is also obtained in the presence of the GCCCU°GG sequence, with an interaction between the E51 carboxylate and N1 atom of 8-oxoG14 (see Supplementary Figure S4B).

**FIGURE 7:**
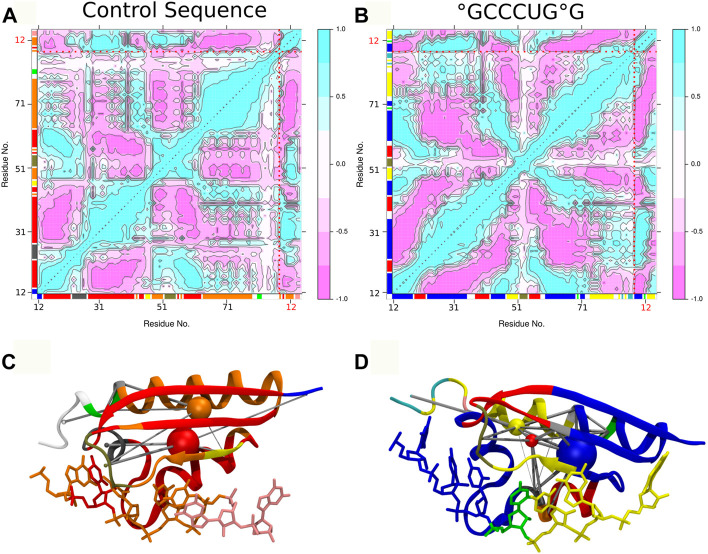
Dynamic cross correlation of residue pairs and corresponding dynamic networks for control **(A,C)** and °GCCCUG°G **(B,D)** sequences. In both graphs, the dotted red lines indicate the separation of the protein and 9 to 15 RNA residues and the axis color corresponding to the network community of each residue. In the 3D representation of networks, the residues are colored according to their dynamic community represented by nodes (colored spheres) connected by the edges (gray cylinders), according to the dynamic cross correlation between them.

The comparative analysis of the dynamic cross correlations of the PCBP1–RNA system confirms the existence of different interaction patterns between the control and the °GCCCUG°G sequences (see Figure 7). In both cases, a strong positive correlation is present between the RNA strand and the protein, but the involved residues differ: in the control sequence simulation, residues 28 to 42 show a good correlation with RNA nucleobases 9 to 13, while G14 and G15 interact more with protein residues 48–51; in the presence of a damage, the first part of the DNA strand has a strong positive correlation with the residues 23 to 35 and the final guanines with 12 to 18 and 48 to 51. Furthermore, a positive correlation exists between the protein residues within the *β*-sheet. Then, different community nodes can be drawn, including both nucleobases and amino acids which share correlated motions. In the control sequence, G9 belongs to the same node as protein residues 48 to 51, but G14 and G15 constitute an independent node with few connections with others. On the contrary, the damaged 8-oxoG9 lesion is included in the same community as K23, residues 12 to 18, 43 to 47, and 60 to 69 (blue node), while the yellow node contains 8-oxoG15 and residues 48 to 51. These two nodes are strongly and directly connected. However, the second part of the loop (residues 52–55) belongs to its own independent node, connected to the *β*-sheet residues.

### Impact of K23 and K31 mutations

In order to assess the role of some protein residues in RNA binding, we perform MD simulations of different mutated PCBP1 KH 1 domains with the targeted sequence °GCCCUG°G. Our previous results suggest that K23 can play a strong role in the preference of PCBP1 for the oxidized RNA sequence. Two mutants are considered: K23Q, which keeps a polar characteristic associated with a relatively long chain, and K23M, where the polar properties are lost, but a relative linear long side chain is kept. In addition, the double mutants K31D and K32D experimentally show a decrease in RNA binding, supporting the idea of a role of these two residues in the protein–RNA interaction (Ishii et al., 2018). In our previous simulation, only K31 presents a remarkable contribution to the binding energy. Consequently, we here focus on the single mutant K31D to limit the impact on protein conformation.

We first investigate the RNA strand behavior. Apart from K23M, the mutations do not impact the inter base or backbone parameters (see Supplementary Figures S5,S6). The lysine-to-methionine mutations mostly impact the 8-oxoG15 behavior by increasing its mobility, as suggested by the larger standard deviation and the large distance between its O8 atom and its phosphate group (Supplementary Figure S7B). In terms of the radial distribution function, 8-oxoG15 is similar for all the mutants, whereas only the K23M mutant allow to increase the number of water molecules around the O8 atom of 8-oxoG9. However, the corresponding profile does not present the peak at 2.7 Å and draw a less-structured first hydration shell around the damage.

**FIGURE 8:**
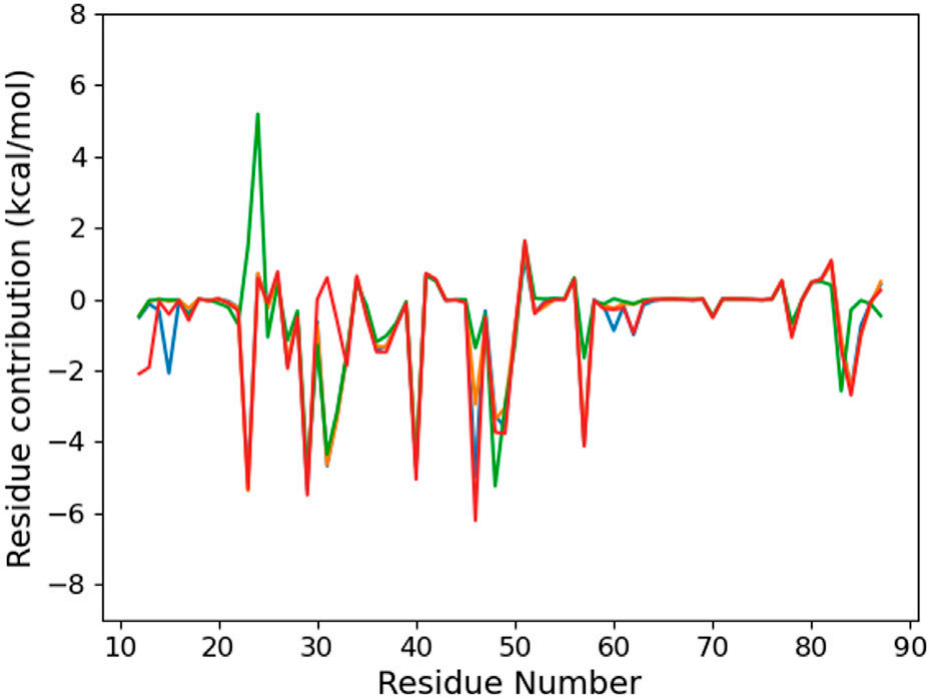
Per-protein residue contribution to the binding energy of the RNA fragment °GCCCUG°G RNA sequence for the different PCBP1 mutants: WT (orange), K23Q (blue), K23M (green), and K31D (red).

The MMPBSA binding energy (Table 2) suggests that the K23Q mutation does not affect the RNA–protein interaction, whereas the K23M and K31D mutations weaken it by 7–20 kcal/mol compared to WT. The per-residue analysis (Figure 8) confirms that the K23Q mutation does not impact the RNA–protein profile, whereas K23M not only leads to the loss of the 23rd protein residue contribution but also to the appearance of a strong repulsive contribution from E24. Indeed, as we replaced lysine with glutamine starting from an equilibrated structure of WT with the °GCCCUG°G sequence, the amino group of the Q23 backbone occupies exactly the same position as the lysine ammonium group and plays the role of the H-bond bridge between the 8-oxoG O8 atom and the phosphate of C10 (see Figure 10C). In the K23M mutant, methionine does not interact with RNA and recovers a flexible behavior, far from the nucleobases (see Figure 10D). The aspartate in position 31 does not interact with the RNA sequence, but this mutation does not impact the per-residue contribution profile elsewhere.

**TABLE 2:** Average and standard deviations (in kcal/mol) of the binding energy of the RNA fragment °GCCCUG°G to the PCBP1 RNA-binding domain for the different protein mutants K23Q, K23M, and K31D. The values at different times along the 1-*μ*s trajectories.

Time (*μ*s)	WT	K23Q	K23M	K31D
0.5–0.6	−26.4 ± 8.4	−26.8 ± 8.8	−12.4 ± 7.4	−4.6 ± 7.8
0.7–0.8	−24.8 ± 7.3	−20.6 ± 8.2	−17.3 ± 8.5	−7.9 ± 7.2
0.9–1	−25.5 ± 8.1	−26.2 ± 6.6	−6.0 ± 9.9	−18.0 ± 7.6

**FIGURE 9:**
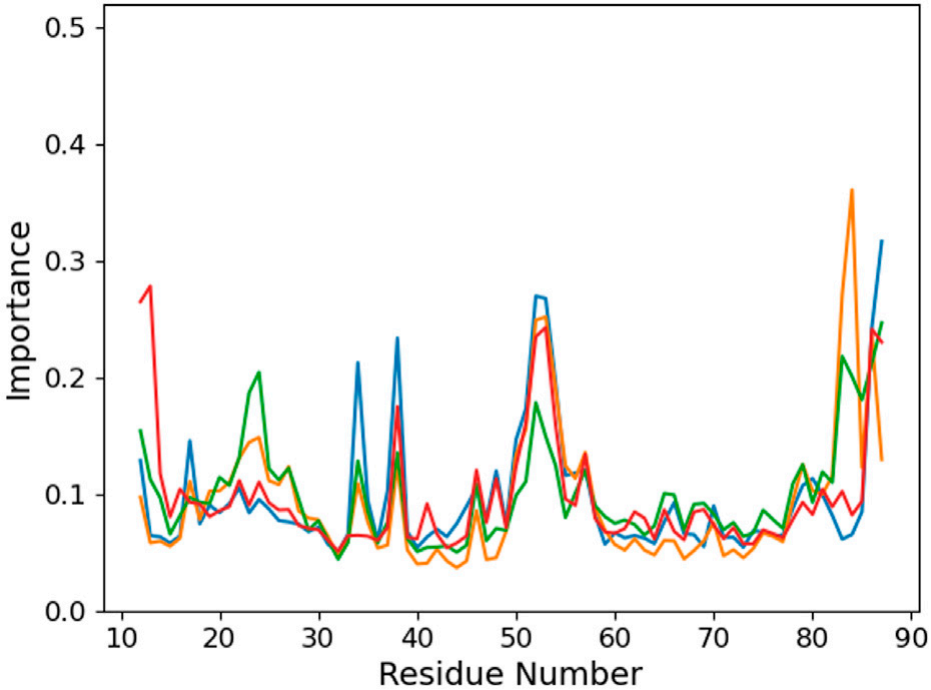
Per-protein residue importance from the PCA analysis for the different PCBP1 mutants in interaction with the °GCCCUG°G RNA sequences: WT (orange), K23Q (blue), K23M (green), and K31D (red).

**FIGURE 10:**
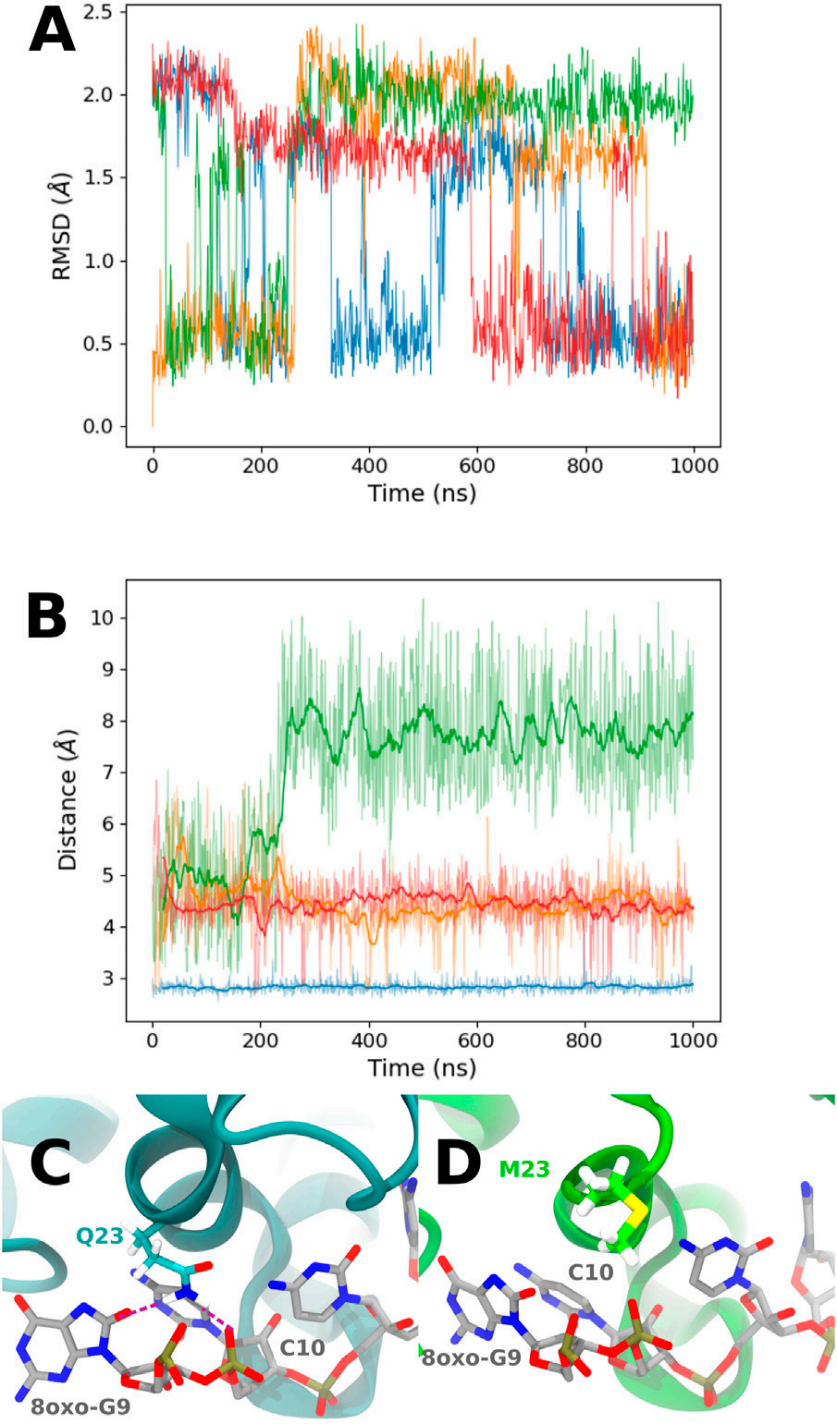
**(A)** RMSD of the C*α* atoms of the 49–55 PCBP1 residues in the presence of the °GCCCUG°G RNA sequence for the different PCBP1 mutants: WT (orange), K23Q (blue), K23M (green), and K31D (red). **(B)** Time evolution of the distance between 8-oxoG9 O8 and K23 N*ζ* for WT (orange) and K31D mutants (red), 8oxoG9 O8 and Q23 N*ϵ* for the K23Q mutant (blue), or 8oxoG9 O8 and M23 C*ϵ* for the K23M mutant. **(C)** Q23 interaction with 8-oxoG9 O8 and C10 phosphate. Interactions are represented with purple dashed lines. **(D)** Example of a M23 conformation in the K23M mutant.

Looking at the PCA profiles (Figure 9), we can observe a decrease in the relative importance of the 19–28 protein sequence when K23 is mutated in glutamine or when K31 is mutated to aspartate. On the contrary, the K23M mutation increases the importance of residues 23 and 24, suggesting that the disappearance of the long side chain of lysine allows more conformational rearrangement that does not favor RNA binding. Moreover, this mutation is the only one from our set that impacts the importance of the 49–55 loop by decreasing it. Intriguingly, no open conformation of this loop is observed for K23Q, but it quickly reaches the K23M simulation, where the interaction between residue 23 and RNA is lost (Figures 10A,B). The loop of the K31D mutant starts in an open geometry to move to the closer one after 600 ns. Looking at other distances (Supplementary Figures S4C,F), we observe that the S50-G14 interaction is associated with the open conformation of the loop, while the T15-8oxoG15 hydrogen bond can be formed even in the close conformation in the K23Q and K31D mutants and comes to strengthen the RNA binding, as suggested by the −2 kcal/mol contribution of T15 to the protein–RNA binding energy (Figure 8).

## Discussion

We herein investigate the RNA binding by the PCBP1 KH 1 domain in the presence of 8-oxoG by means of MD simulations to characterize molecular mechanisms that explain the protein preference for severely oxidized RNA sequences. Several sequences have been considered consistently with experiments, with different positions for the 8-oxoG lesion. Although no unbinding event has been observed due to the strong interaction between the CCC trimer and the protein in our microsecond timescale MD trajectories, our binding free energy analysis is consistent with the experiment with a clear preference for the °GCCCUG°G sequence. Indeed, the contribution of the lysine K23, which comes to interact with phosphate groups of bases at positions 9 and 10 and the O8 atom of 8-oxoG9, strengthens the whole RNA binding. This interaction relies on the modified polar properties of 8-oxoG compared to the undamaged guanine: the extra ketone group is prone to form a hydrogen bond with a bridging molecule that also interacts with the RNA backbone. The latter molecule can be a water molecule (as observed for 8-oxoG in positions 13, 14, or 15) or a polar residue such as lysine. This interaction also impacts the rigidity of the RNA strand. Nevertheless, this K23–RNA interaction is not observed when no other 8-oxoG is present or if it is too close (at position 13). This different behavior suggests that 8-oxoG in position 15 is required to trigger this interaction, whereas this damaged guanine does not seem to have a specific interaction with the protein. Indeed, only few hydrogen bonds have been observed between the purine nitrogen atoms and some threonines located in the PCBP1 *β*-sheet. However, the machine-learning principal component analysis reveals the importance of the 49–55 loop, standing between *β*-sheet strands and relatively close to K23. During the simulation in the presence of the °GCCCUG°G sequence, this loop moves from a close to an open conformation, which is concomitant with the formation of the K23–RNA interaction. Then, the loop retrieves its close conformation, but K23 still interacts with the RNA backbone. Our results suggest an allosteric mechanism, involving the transient fixation site of 8-oxoG15 and the motion of K23 close to 8-oxoG9, with a communication through the *β*-sheet and the 49–55 loop. The strong dynamic cross correlation between the different parts of RNA and the *β*-sheet present for the damaged sequence but absent in the control simulation, especially around G15, tends to confirm this hypothesis. To assess the role played by K23, we have computationally mutated K23 to glutamine and methionine. In the first case, the amine group is able to replace the ammonium group and thus conserve the interaction with the RNA backbone and the 8-oxoG9 O8 atom. In the second case, the interaction is lost as methionine has no polar group, and the binding free energy decreases by about 20 kcal/mol. We have also tested the K31D mutation, inspired by the experiment. Combined to the K32D mutation, it experimentally impedes the severely damaged sequence specific recognition by PCBP1. In our simulation, this mutation induces the loss of the electrostatic interaction between lysine and the RNA backbone but does not impact the 8-oxoG9 binding. As this interaction is not specific to the oxidized RNA sequence, our results are consistent with a participation of K31 to the upstream recognition mechanism and not to the specific stabilization of the oxidized RNA–PCBP1 interaction. This work also illustrates the current performance of GPU-accelerated molecular dynamics simulations to capture fine sequence effects for RNA or DNA repair (Gattuso et al., 2016).

## Data Availability

The raw data supporting the conclusion of this article will be made available by the authors, without undue reservation.
