# Supplementary material for: Dynamics and energetics of PCBP1 binding to severely oxidized RNA
Source: Front Mol Biosci. 2022 Nov 3;9:994915. doi: 10.3389/fmolb.2022.994915 (PMC9671708; doi:10.3389/fmolb.2022.994915)
Supplement: Supplementary file 1 [file DataSheet1.PDF]

# Supplementary Material

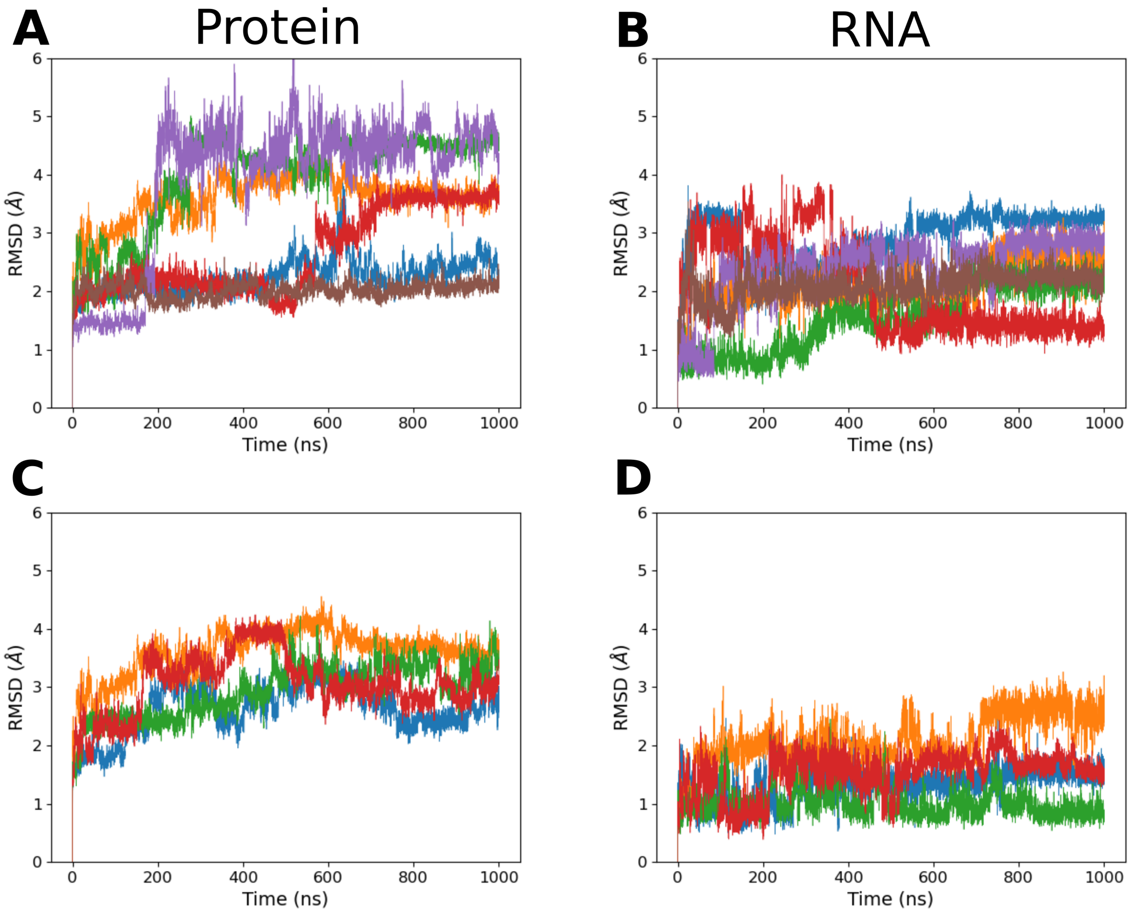


**Figure S1.** RMSD time evolution for protein (**A** and **C**) and 9-15 RNA sequence (**B** and **D**) along the 1 $\mu$s trajectory for the RNA sequences (**A** and **B**): control (blue), °GCCCUG°G (orange), GCCCU°GG (green), °GCCCUGG (red), GCCCUG°G (violet), °GCCC°GGG (brown); or the protein variant in presence of °GCCCUG°G RNA sequence: WT (orange), K23Q (blue), K23M (green), K31D (red). Structures where align on the first protein position.

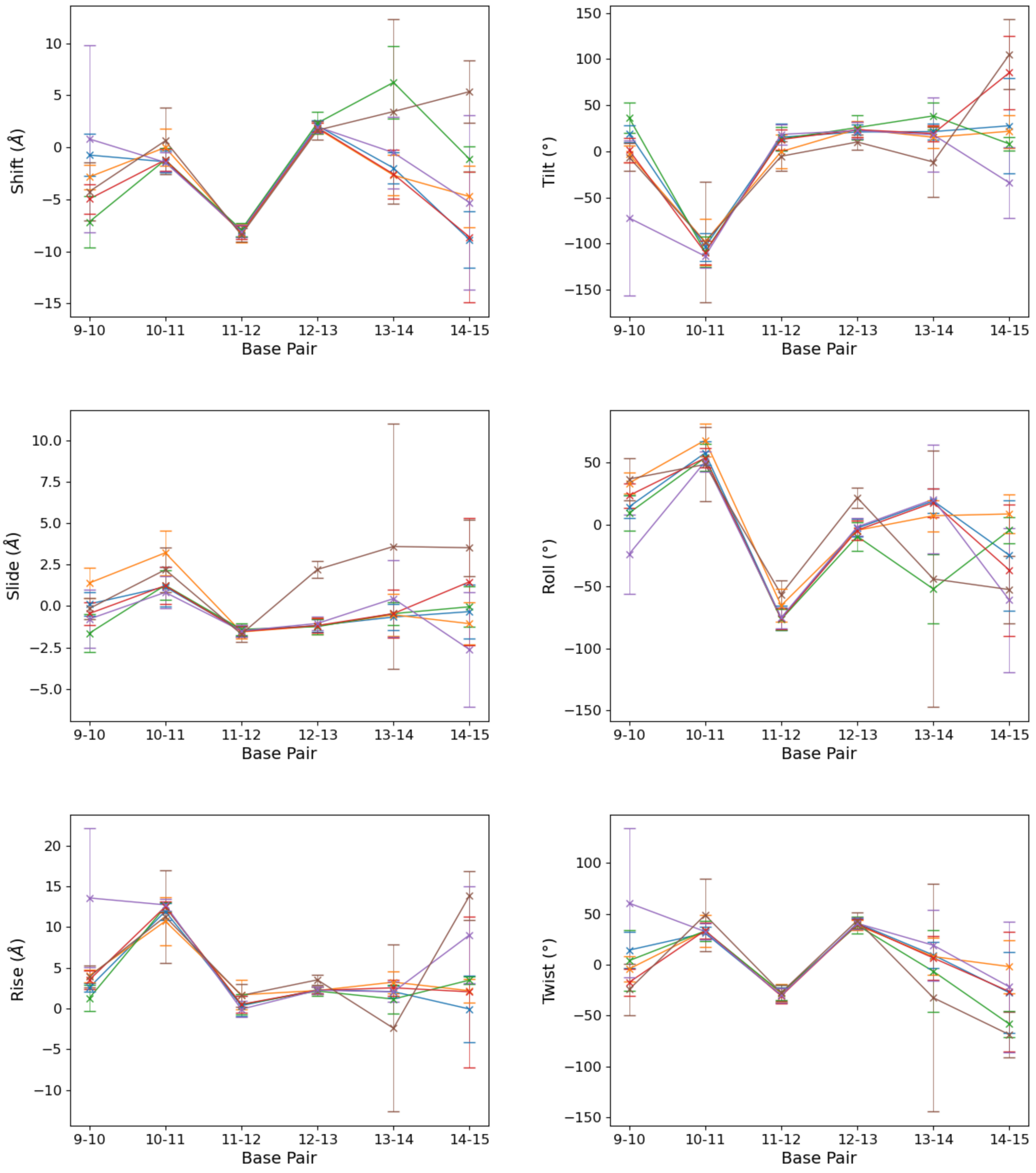


**Figure S2.** Mean RNA inter bases parameters for the different RNA sequences: control (blue), °GCCCUG°G (orange), GCCCU°GG (green), °GCCCUGG (red), GCCCUG°G (violet), °GCCC°GGG (brown). Errors bars correspond to standard deviations.

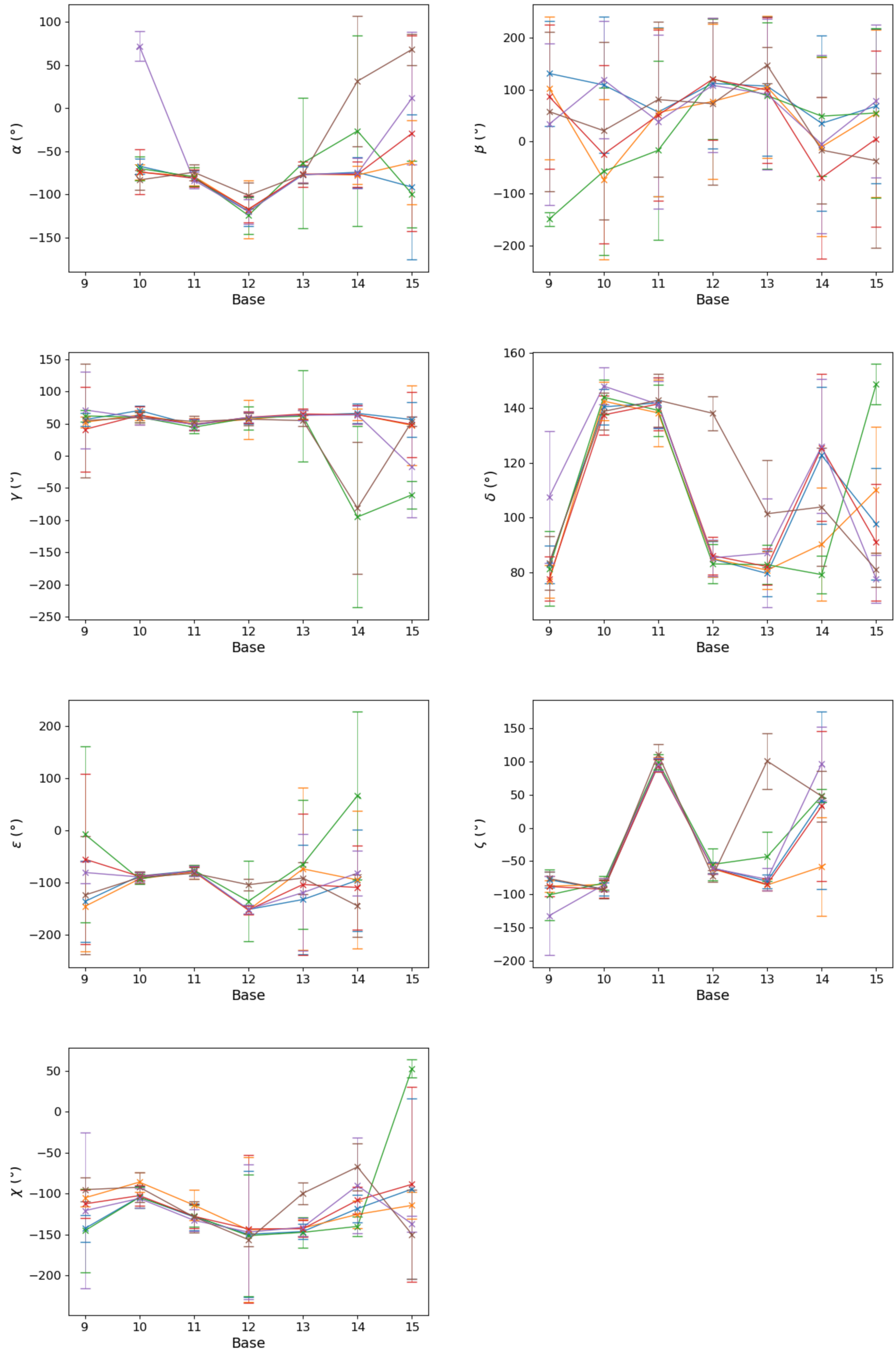


**Figure S3.** Mean RNA backbone dihedral angles for the different RNA sequences: control (blue), °GCCCUG°G (orange), GCCCU°GG (green), °GCCCUGG (red), GCCCUG°G (violet), °GCCC°GGG (brown). Errors bars correspond to standard deviations.

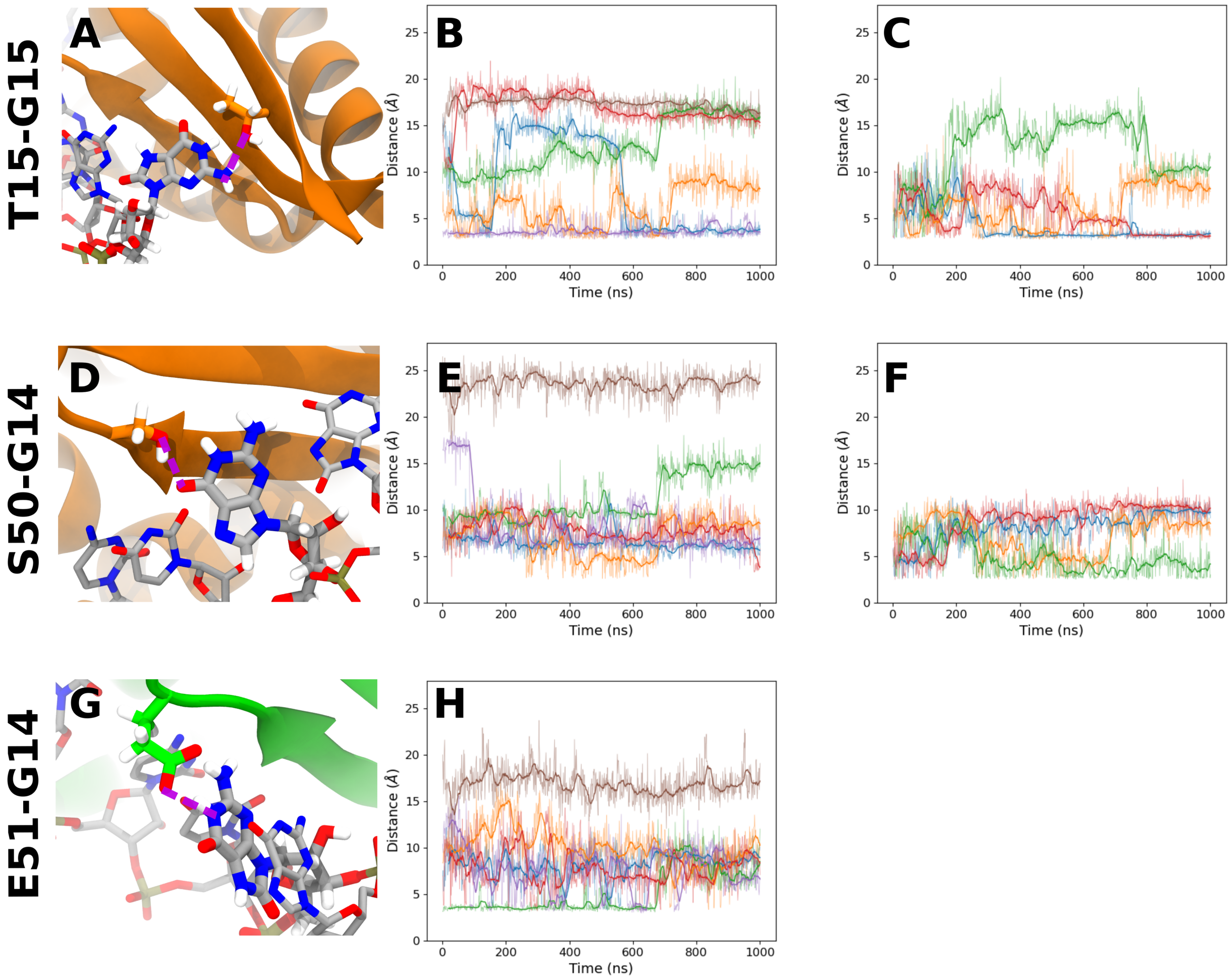


**Figure S4.** Representation (**A**, **D**, **G**) and time evolution of the different distances for interaction involving atom T15 O$\gamma$ and G15 (or °G15) N2 (**A**, **B** and **C**), S50 O$\gamma$ and G14 (or °G14) O6 (**D**, **E** and **F**) and E51 C$\delta$ and G14 (or °G14) N1 (**G** and **H**). **B**, **E** and **H** report the data for the different RNA sequences: control (blue), °GCCCUG°G (orange), GCCCU°GG (green), °GCCCUGG (red), GCCCUG°G (violet), °GCCC°GGG (brown); and **C** and **F** for the different protein mutants WT (orange), K23Q (blue), K23M (green), K31D (red).

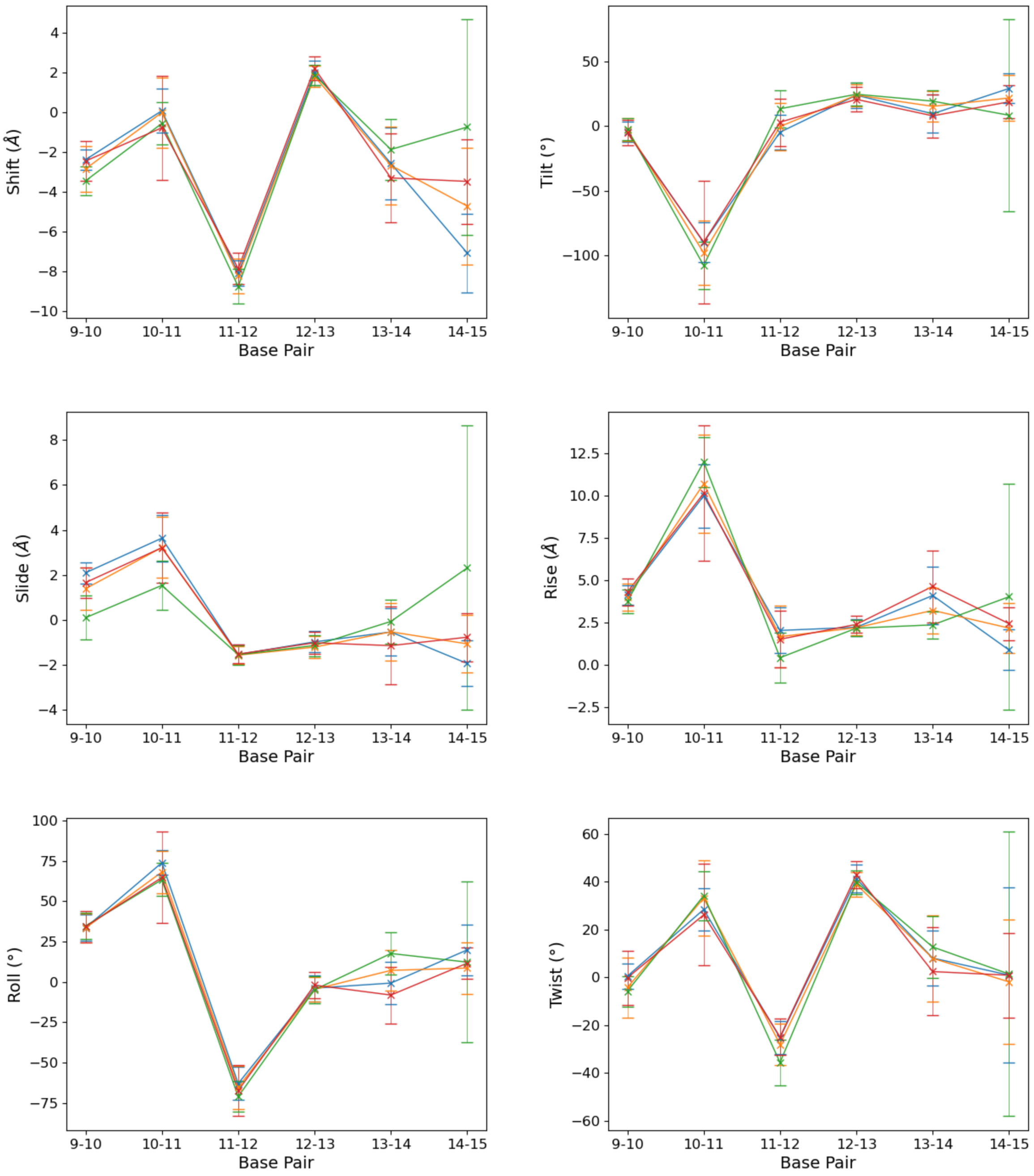


**Figure S5.** Mean RNA inter bases parameters for the different PCBP1 mutant in the presence of the °GCCCUG°G RNA sequence: WT (orange), K23Q (blue), K23M (green), K31D (red). Errors bars correspond to standard deviations.

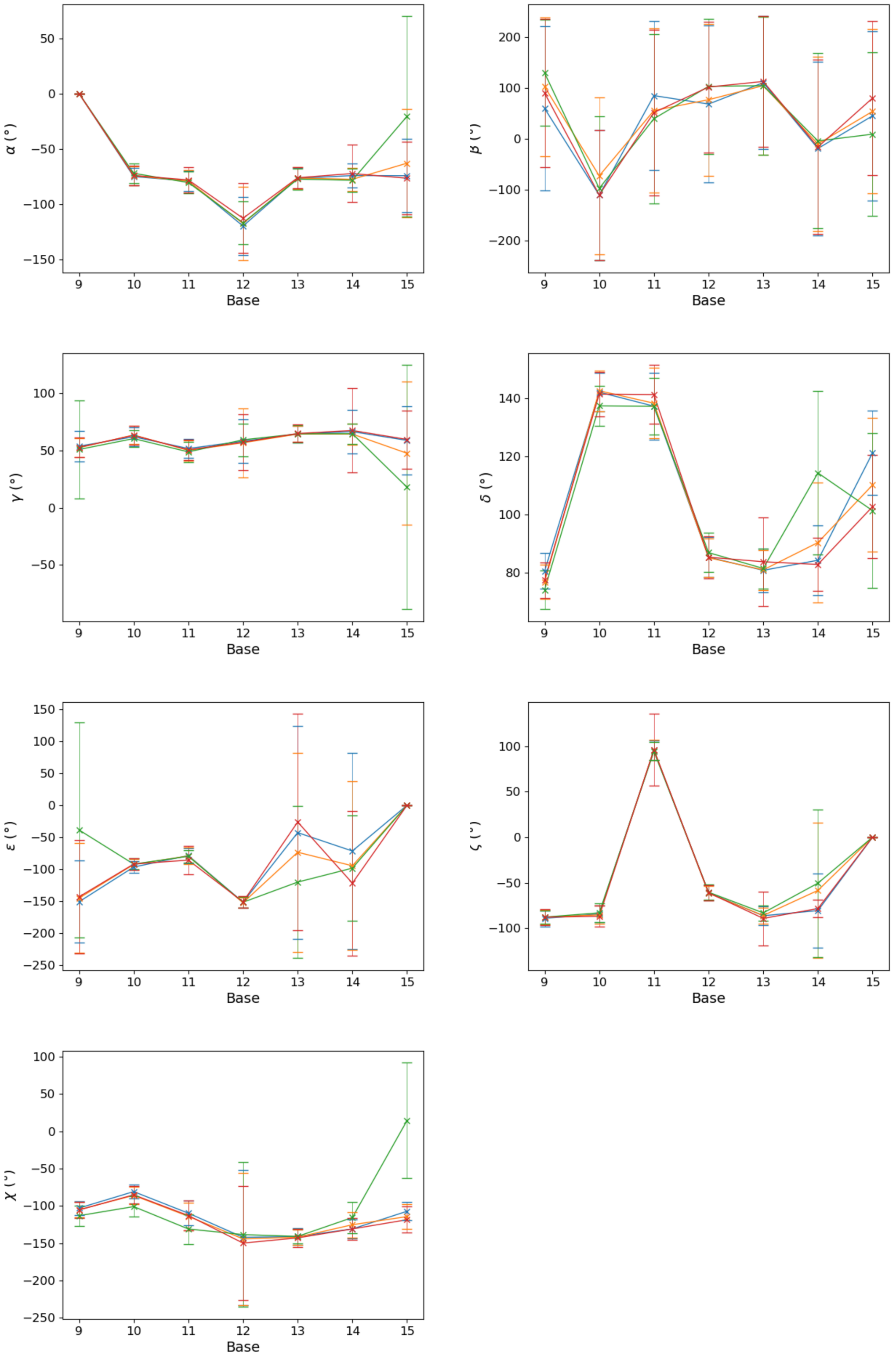


**Figure S6.** Mean RNA backbone dihedral angles for the different PCBP1 mutant in the presence of the °GCCCUG°G RNA sequence: WT (orange), K23Q (blue), K23M (green), K31D (red). Errors bars correspond to standard deviations.

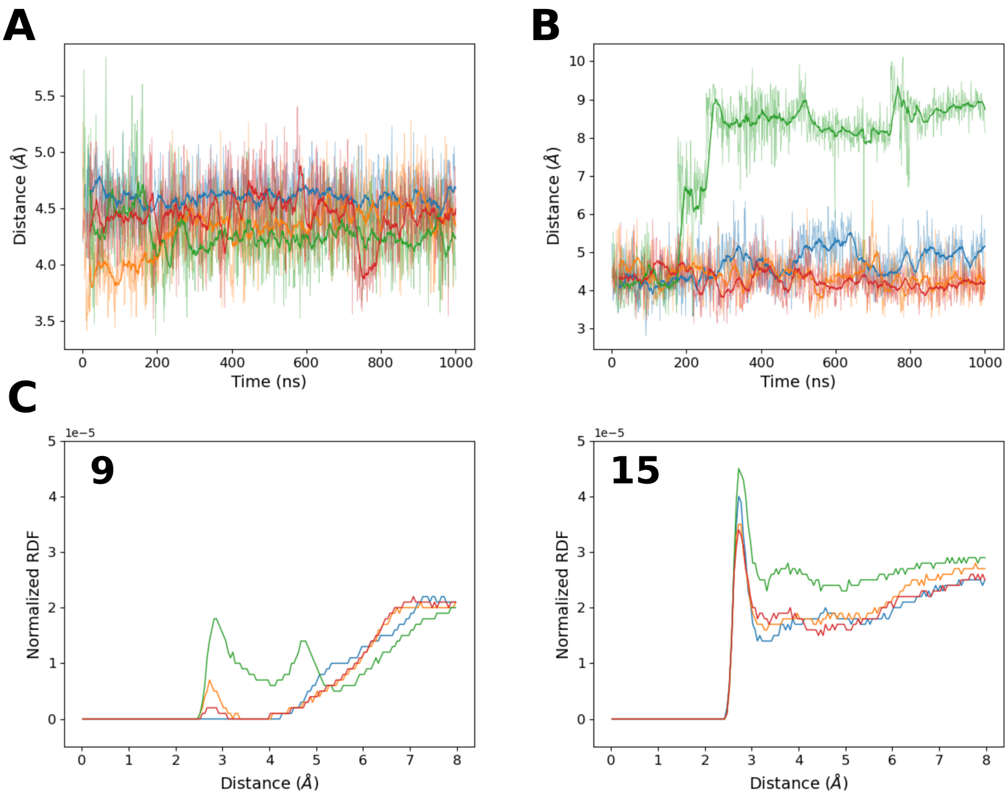


**Figure S7.** **A**. Time evolution of the distance between 8-oxoG9 O8 and the closest 8-oxoG9 phosphate oxygen atom for the RNA °GCCCUG°G sequences with different protein mutants.**B** Time evolution of the distance between 8-oxoG O8 and the closest 8-oxoG phosphate oxygen atom for damage at the 15th position for the RNA °GCCCUG°G sequences with different protein mutants. **C**. Radial distribution function of water oxygen atom around the O8 atom of the different 8-oxoG in position 9 and 15 for the RNA °GCCCUG°G sequences with different protein mutants. For all graphs, the colors are: WT (orange), K23Q (blue), K23M (green), K31D (red).
